# Effects of Different Types of Interlayers on the Interfacial Reaction Mechanism at the Cu Side of Al/Cu Lap Joints Obtained by Laser Welding/Brazing

**DOI:** 10.3390/ma14247797

**Published:** 2021-12-16

**Authors:** Ruican Zhu, Shixiong Guo, Chao Huang, Zhenglong Lei, Xinrui Zhang, Jinge Liu

**Affiliations:** 1 Capital Aerospace Machinery Corporation Limited, Beijing 100076, China; zhurc_211@163.com (R.Z.); shahuhu0126@163.com (S.G.); huangchao881022@163.com (C.H.); 2 Department of Mechanical Engineering, Tsinghua University, Beijing 100084, China; 3 State Key Laboratory of Advanced Welding and Joining, Harbin Institute of Technology, Harbin 150001, China; 20b909125@stu.hit.edu.cn (X.Z.); ljg@163.com (J.L.)

**Keywords:** Al/Cu lap joint, laser welding/brazing, tin foil, Ni coating, interfacial microstructure, interfacial bonding mechanism

## Abstract

The influence of tin foil and Ni coatings on microstructures, mechanical properties, and the interfacial reaction mechanism was investigated during laser welding/brazing of Al/Cu lap joints. In the presence of a Zn-based filler, tin foil as well as Ni coating strengthened the Al/Cu joints. The tin foil only slightly influenced the joint strength. It considerably improved the spreading/wetting ability of the weld filler; however, it weakened the bonding between the seam and the Al base metal. The Ni coating considerably strengthened the Al/Cu lap joints; the highest tensile strength was 171 MPa, which was higher by 15.5% than that of a joint without any interlayer. Microstructure analysis revealed that composite layers of Ni_3_Zn_14_–(τ_2_ Zn–Ni–Al ternary phase)–(α-Zn solid solution)–Al_3_Ni formed at the fusion zone (FZ)/Cu interface. Based on the inferences about the microstructures at the interfaces, thermodynamic results were calculated to analyze the interfacial reaction mechanism. The diffusion of Cu was limited by the Ni coating and the mutual attraction between the Al and Ni atoms. The microstructure comprised Zn, Ni, and Al, and they replaced the brittle Cu–Zn intermetallic compounds, successfully strengthening the bonding of the FZ/Cu interface.

## 1. Introduction

Copper is widely used in various industries such as refrigeration, electrical, building, coinage, and transport owing to its excellent thermal and electrical conductivities [1]. Hai-lat. M and Laherrere expressed concerns about the potential scarcity in production due to the excessive consumption of copper and limited copper reserves [1,2]. Thus, to ensure cost efficiency and development of light materials, it is necessary to replace copper or reduce its usage. Aluminum and copper have similar thermal and electrical conductivities, machinability, and corrosion resistance; in addition, the density of aluminum is lower and its reserves are more abundant compared with those of copper [3,4]. Hence, aluminum can replace copper to some extent, and aluminum–copper hybrid structures are a feasible way to broaden the range of these applications. Al/Cu dissimilar joints have future-orientated applications in various fields such as refrigeration, transportation, high-capacity batteries, and aerospace [5]. Studies have focused particularly on developing Al/Cu dissimilar joints for such applications. However, there is a considerable difference in the melting points of copper (1356 K) and aluminum (933 K), which makes it challenging to join the two using fusion welding because severe burning loss of aluminum occurs while copper just starts to melt [6,7]. In addition, the differences in the thermal expansivities of Al and Cu and the formation of brittle metallic compounds at the joints cause huge residual stresses and cracks [7,8,9,10].

Various welding methods have been investigated to join aluminum and copper such as explosive welding [8,11,12], friction stir welding (FSW) [10,13,14,15], electron beam welding (EBW) [16], magnetic pulse welding [17,18,19], diffusion bonding [20,21], cold roll welding [22], laser welding [1,23], and brazing [24,25,26]. These studies have mostly focused on two ways for optimizing layers of intermetallic compounds (IMCs), namely, adding other elements to the metallurgical reaction system to avoid brittle IMC phases, such as θ (Al_2_Cu)), η (AlCu), or γ (Al_4_Cu_9_) phases [27], and limiting the thicknesses of IMC layers by optimizing process parameters. In EBW, α-Cu layers and brittle phases (Al_4_Cu_9_ and Al_2_Cu) were observed at the weld/Cu interface; however, the uncontrolled formation of brittle phases could be avoided by controlling the heat input via controlling the energy of the electron beam. Cracks started in the IMC layer (tensile strength = 104 MPa). For the Al/Cu lap joints obtained in the laser welding process, the tensile shear strength increased with increasing welding speed. The formation of the IMC layer could be suppressed, and the thickness of the IMC layer decreased to approximately 5 μm at a welding speed of 50 m·min^−1^ [23]. In the FSW process, IMC layer thickness could be controlled by controlling the heat input (very thin IMC layer for low heat input). P. Xue et al. [28] obtained sound defect-free joints with a thin, uniform, continuous IMC layer by optimizing process parameters, and the maximum ultimate tensile strength was 110 MPa. They also found that a sufficient metallurgical reaction between Al and Cu was necessary to ensure high joint strength. Thus, the thickness of the IMC layer should be optimal and neither too thin nor too thick [29]. 

Researchers have added alloying elements such as Ni, Ti, Zn, Sn, Si, and Ag to the Al–Cu binary reaction system [1,5,24,25,26]. Ihor Mys et al. [30] used Ni, Ag, and Sn galvanic coatings and filler interlayers on copper surfaces and to join Al and Cu, respectively. Compared with joints without coatings, the application of three coatings, especially of Ag and Sn coatings, improved the tensile strength of the Al/Cu joint. Use of a Ni foil filler led to an only negligible improvement of the strength and a slight reduction of the spreading width, which was attributed to the formation of Al–Ni IMCs; the low thermal expansion of Al–Ni IMCs increased the residual stresses. In case of Sn and Ag foils, joint strength enhancement was attributed to the formation of a ductile ternary phase. Sahu et al. [5] joined Al/Cu using FSW with Ni, Ti, and Zn foils. Ni, Ti, and Zn reacted with the Al and Cu base metals and formed Al_7_Cu_4_Ni, Ti_3_Al, and CuZn_5_ IMCs, respectively. The foils enhanced the joint strength to some extent but decreased the ductility. Zn foil provided the most optimal results. Huang et al. [24,25] brazed Al/Cu dissimilar joints using Al/Sn–9Zn–xAg/Cu and Al/Sn–9Zn–xNi/Cu solders. The strength decreased with increasing amounts of Ag and Ni due to the negative effects of IMCs produced during the process, such as AgZn_3_, Ni_3_Sn_4_, and Al_3_Ni. Hailat et al. [1] produced Al/Cu joints using laser welding; they used tin foil interlayers to prevent the formation of Al–Cu IMCs, which increased the shear strength and elongation of the joint. 

In all the aforementioned cases, the common concern is the formation of IMCs in Al/Cu joints. Controlling the heat input during the welding process and adding elements to the reaction system were effective ways to limit the growth of Al–Cu IMC layers. In this study, laser welding/brazing (LWB) was used to produce Al/Cu joints. Researchers often offset the laser beam to control the melted amount of the base metals [31,32,33], and the formation of the IMCs could be inhibited. In this research, copper has a high melting point and low light absorption. Laser beam offset to the Cu sheet, the heat input was adjusted so that the Al base metal melted while Cu did not melt, thus avoiding the excessive mixing of Al and Cu. The low heat input also suppressed the reaction in the thermal cycle. Moreover, a Zn–2Al filler, tin foil, and Ni coating were employed to improve the mechanical properties of the joint. The low Al content in the Zn-based filler limited the formation of brittle Al–Cu IMCs [7,24,25,34].

Thin and uniform Cu–Zn IMCs replaced the Al–Cu IMCs at the interface and limited the interfacial strength around the Cu sheet [35]. Along with the Zn-based filler, Sn and Ni were added at the interface between the seam and the copper sheet for further enhancement of the joint, and the joint appearance, interfacial microstructure, and mechanical properties were investigated. To further examine the strengthening effect of the Ni coating on the strength of the interface, thermodynamic calculations were carried out and the bonding mechanism was clarified in detail.

## 2. Experimental Design

### 2.1. Material and LWB Process

The base metals were 6061 aluminum alloy and H62 copper alloy sheets; specimens with same sizes were used (100 mm × 50 mm × 1.8 mm). A Zn–2Al weld was used as the filler. The chemical compositions of the base metals and the filler are listed in Table 1 [7,36]. Tin foil (varying thicknesses) was used in the study; these values and the process parameters are listed in Table 2. These values and the process parameters are listed in Table 2. Based on the experimental study on the joining process of Al/Cu dissimilar alloys, we chose the optimal process parameters when a Zn-based filler was used; the same parameters were adopted when adding Sn and Ni interlayers.

**Figure 1:**
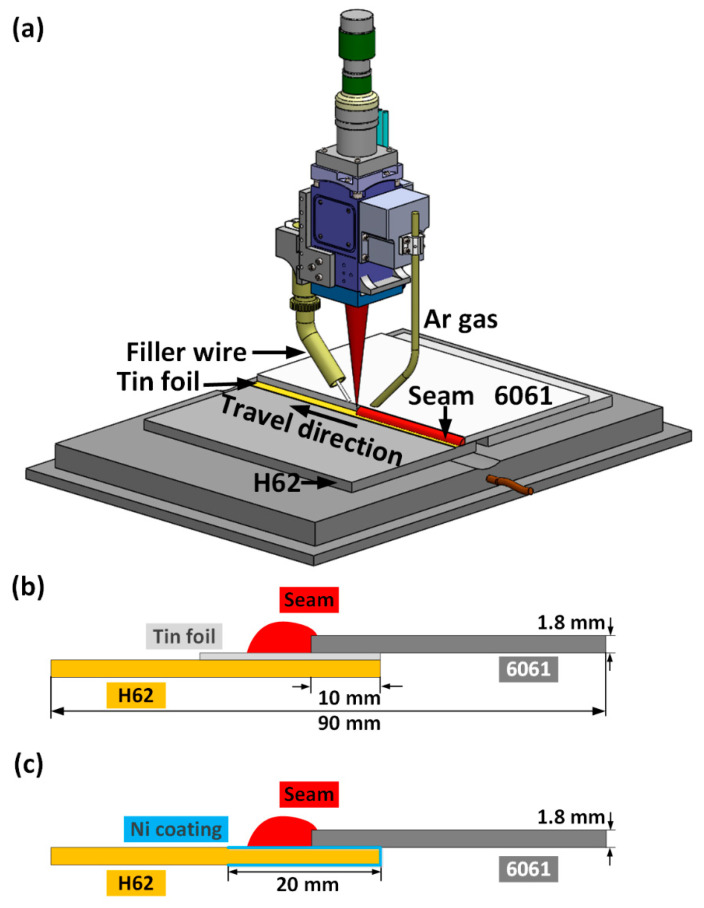
Schematic of LWB Al/Cu dissimilar joints: (**a**) LWB process. Reprinted with permission from Ref. [31] copyright 2021 Elsevier B.V.; (**b**) size and schematic of a lap joint with tin foil; (**c**) size and schematic of an Al/Ni-coated Cu lap joint.

The LWB process is schematically illustrated in Figure 1a [31]. A fiber laser (5000 W, wavelength = 1070 nm, IPG YLR-5000, IPG Inc., MA, USA) was used. The diameter of the focused laser beam was 1.2 mm with a beam parameter product of 7.2 mm × mrad. The laser incident direction was deviated from the normal of the workpiece surface (5°) to avoid burnout of the device. Both base metals were ground using a grinder to clean any oxidation film and grease on their surfaces before welding. The lap joint configuration with tin foil is shown in Figure 1b. The Al sheet was placed on top of the copper sheet (lap length = 10 mm). The tin foil with varying thicknesses was sandwiched between the two sheets. Figure 1c shows the joint configuration after addition of Ni. Ni was electroplated on the cleaned H62 copper sheets using an electrochemical method. The maximum thickness of the Ni coating was 20 mm on the H62 copper sheets. The optimal thickness of the Ni coating was determined to be 4.56 μm by controlling the electroplating time.

### 2.2. Analysis Methods

After the LWB process, four test specimens were extracted for tensile strength tests and metallographic analyses. One specimen was polished using emery papers, and a canvas and was used for macro- and microstructure analyses. Three specimens were used for tensile strength tests. Metallographic analysis of weld cross-sections was carried out using optical microscopy. The samples were not etched. Phase analysis at the interface was performed via the back-scattered electron (BSE) mode of scanning electron microscopy (SEM) equipped with energy-dispersive spectrometry (EDS). X-ray diffraction (XRD) was used to determine the phase chemical composition by observing EDS profiles. For tensile strength tests, 90 mm × 6 mm specimens were used. The loading speed was 0.5 mm/min at room temperature. The final value of the ultimate tensile strength was the average value of the tensile strength of the three specimens.

### 2.3. Thermodynamic Analysis

Thermodynamic analyses were carried out to examine the metallurgical reaction. Miedema’s model was used for calculating formation enthalpy in the binary system [37].

(1) $$\Delta H_{1,2}=f_{1,2}\frac{x_{1}[1+\mu_{1}x_{2}(\varphi_{1}-\varphi_{2})]x_{2}[1+\mu_{2}x_{1}(\varphi_{2}-\varphi_{1})]}{x_{1}V_{1}^{2/3}[1+\mu_{1}x_{2}(\varphi_{1}-\varphi_{2})]+x_{2}V_{2}^{2/3}[1+\mu_{2}x_{1}(\varphi_{2}-\varphi_{1})]}$$

(2) $$f_{1,2}=\frac{2pV_{1}^{2/3}V_{2}^{2/3}[q/p{\left(\Delta n_{WS}^{1/3}\right)}^{2}-{\left(\Delta \varphi \right)}^{2}-\alpha (r/p)]}{{\left(\Delta n_{WS}^{1/3}\right)}_{1}{}^{-1}+{\left(\Delta n_{WS}^{1/3}\right)}_{2}{}^{-1}}$$

where Δ*H* is the enthalpy change in the reaction of the binary system, *x*_1_ and *x*_2_ are the molar fractions in the system, *φ* is the electronegativity, *V* is the molar volume, and *n_WS_* is the electron density; *μ*, *p*, *q*, *r*, and *α* are experimental constants in Miedema’s model. 

Based on Miedema’s model, a quaternary Toop model was developed. In this study, the quaternary model was used because four elements were considered. The following equations [38,39] were used for calculating the Gibbs free energy, *G^E^*, and the chemical potential, *μ_i_*:

(3) $$\begin{array}{l} G^{E}=\frac{x_{2}}{1-x_{1}}G_{12}^{E}(x_{1},1-x_{1})+\frac{x_{3}}{1-x_{1}}G_{13}^{E}(x_{1},1-x_{1})+\frac{x_{4}}{1-x_{1}}G_{14}^{E}(x_{1},1-x_{1})+{\left(x_{2}+x_{3}\right)}^{2}G_{23}^{E}(\frac{x_{2}}{x_{2}+x_{3}},\frac{x_{3}}{x_{2}+x_{3}})\\ +{\left(x_{2}+x_{4}\right)}^{2}G_{24}^{E}(\frac{x_{2}}{x_{2}+x_{4}},\frac{x_{4}}{x_{2}+x_{4}})+{\left(x_{3}+x_{4}\right)}^{2}G_{34}^{E}(\frac{x_{3}}{x_{3}+x_{4}},\frac{x_{4}}{x_{3}+x_{4}}) \end{array}$$

(4) $$G_{m}=G^{ID}+G^{E}$$

(5) $$\mu_{i}=\frac{\partial G_{m}}{\partial x_{i}}$$

(6) $$G_{12}^{E}=\Delta H_{1,2}[1-T(1/T_{m,1}+1/T_{m,2})/14]$$

(7) $$G^{ID}=G^{0}+\Delta G^{ID}=x_{1}G_{1}^{*}+x_{2}G_{2}^{*}+x_{3}G_{3}^{*}+x_{4}G_{4}^{*}+RT(x_{1}\ln x_{1}+x_{2}\ln x_{2}+x_{3}\ln x_{3}+x_{4}\ln x_{4})$$

where $G_{ij}^{E}$ was determined using Tanaka’s theory and *G^ID^* is the Gibbs free energy of the ideal solution approximation, $T_{m,i}$ is the melting point of the element, $G_{i}^{*}$ are the molar free energies, and *R* is the gas constant. Physical parameters of Al, Cu, Zn, and Ni are listed in Table 3 [31,40,41,42].

In this study, to understand the metallurgical reaction at the interface, the interfacial quaternary reaction system was discussed. T is the temperature at the seam and the interface zone. However, it is difficult to determine the exact temperature in the interface zone. Hence, the value of the interfacial temperature was calculated using numerical simulation [31,40]. 

## 3. Result and Discussion

### 3.1. Joint Appearances and Cross-Sections

**Figure 2:**
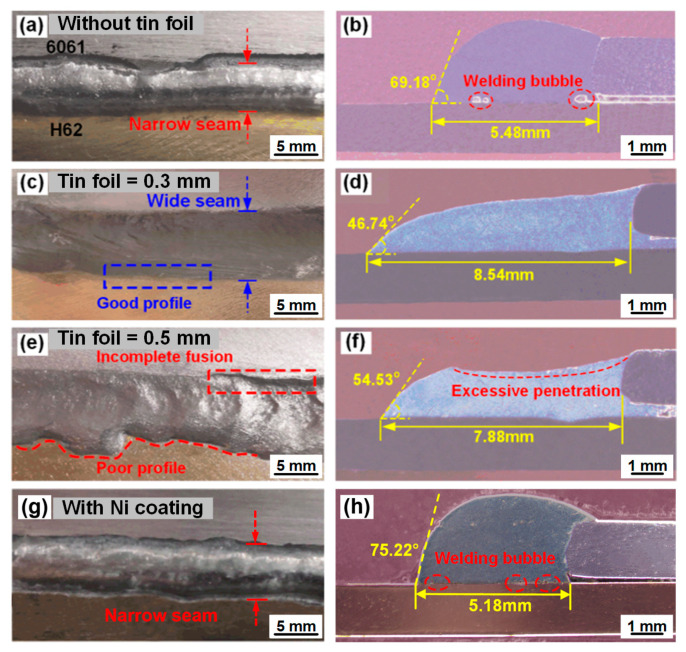
Appearances and cross-sections of LWB dissimilar Al/Cu joints:(**a**) appearance without tin foil [40]; (**b**) cross-section without tin foil [40]; (**c**) appearance with 0.3 mm tin foil; (**d**) cross-section with 0.3 mm tin foil; (**e**) appearance with 0.5 mm tin foil; (**f**) cross-section with 0.5 mm tin foil.

The melting point of tin foil is only 231 °C; because of this, it has good fluidity during the welding process, which leads to improved wetting/spreading ability of filler solders on the surface of the copper sheet. First, the effects of the addition of tin foil on the macroscopic morphology of the welding seam were investigated. Figure 2a,b [40], from our previous research shows the appearance and the cross sections of the joints, respectively, without tin foil. After bonding with tin foil, the joints had wide seams (Figure 2c,e). When the thickness of tin foil was 0.3 mm, the seam was continuous and smooth and showed a good profile (Figure 2c). For tin foil thickness of 0.5 mm, the seam showed a poor profile (Figure 2e) with uneven width and defects due to incomplete fusion. Thus, large thicknesses of tin foil led to unstable spreading, which negatively affected the macro appearance. As seen in Figure 2g, the joint with the Ni coating appeared continuous and stable, but it had a narrow seam.

Figure 2b,d,f,h [40] shows the cross-sections of the joints. The contact angle, θ, and the spreading width of the seam were measured to examine the wetting/spreading ability. The contact angle was 46.74° and 54.53° for tin foil thickness of 0.3 mm and 0.5 mm, and the spreading widths were 8.54 mm and 7.88 mm, respectively. Compared with the Al/Cu joints without tin foil [35,40], with addition of the tin foil, the contact angle, the spreading width, and the wetting/spreading ability were enhanced. Thus, using tin foil can increase the spreading ability of a Zn–2Al filler.

When tin foil thickness increased from 0.3 mm to 0.5 mm, there was a slight decrease of the seam width (0.66 mm) and an increase in the contact angle (7.79°). Hence, the proper thickness of tin foil was 0.3 mm for the Al/Cu joint with the Zn–2Al weld filler.

For the Al/Ni-coated Cu joint, the contact angle was 75.22° and the spreading width was 5.18 mm, showing a slightly reduced wetting and spreading ability compared with the joint with only the Zn–2Al filler. 

Overall, the findings of this study show that, compared with the joint with only a Zn-based filler, tin foil significantly enhanced the wetting ability of the filler, while the Ni coating had only a slight effect on it.

### 3.2. Interfacial Microstructure

#### 3.2.1. Effect of Tin Foil on the Interfacial Microstructure 

The fusion zone (FZ)/Cu interface is often a weak area in Al/Cu joints [26,43] where crack initiation and propagation tend to occur [35,40]. In this study, greater spreading width of the seam at the Cu sheet strengthened the interfacial bonding; because of this, the Al/FZ interface was also a possible weak area. Hence, microstructures at the FZ/Cu and Al/FZ interfaces were investigated at three locations. The corresponding EDS point results are shown in Table 4. One study reported that a high thermal gradient of the molten pool could lead to the formation of various microstructures in different regions even in the same joint [44]. Therefore, three regions, namely, comprising the direct irradiation region, middle region, and the weld toe region, were observed at the FZ/Cu interface. 

**Figure 3:**
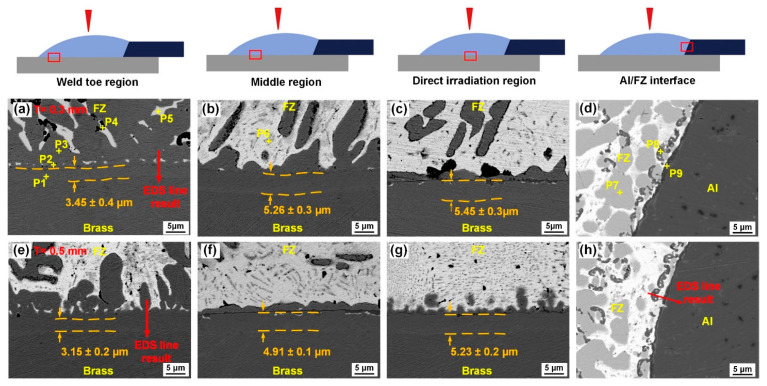
Microstructures of the FZ/Cu and FZ/Al interfaces: (**a**–**d**) joint with 0.3 mm thick tin foil; (**e**–**h**) joint with 0.5 mm tin foil.

Figure 3a–d,e–h shows the interfacial microstructures of the joints with 0.3 mm and 0.5 mm thick tin foils, respectively. Tin foil residue was not observed at the FZ/Cu interface of the joint, indicating that the preset tin foil had completely melted and mixed in the seam. The chemical compositions of the different positions in Figure 3a–d are listed in Table 4.

For the microstructure at the FZ/Cu interface of the Al/Cu joint with a Zn–Al filler, typical multilayers were observed in previous works [7,26,45], such as CuZn + Al_4_Cu_9_ + Al_4.2_Cu_3.2_Zn_0.7_ and CuZn5 + Al_4.2_Cu_3.2_Zn_0.7_. When the Al content of the Zn–Al weld filler adopted in the Al/Cu lap joint was only 2 wt.%, CuZn and CuZn_5_ layers could be observed at the FZ/Cu interface [35,40].

Figure 3a–d shows that a banded layer identified as CuZn (P1 in Figure 3a) is also observed at the FZ/Cu interface. The corresponding EDS results (P2 in Figure 3a) indicate that the Zn content was 88.7 at.%; the Zn content at the P3 position was 89.17 at.%. Microstructures (P2 in Figure 3) adjacent to the CuZn IMC layer and the island-like phase (P3 in Figure 3) in the FZ were both inferred as the α-Zn solid solution. A large amount of α-Al + η-Zn solid solution (P4 in Figure 3) was also observed in the seam near the FZ/Cu interface. Other phases in the seam were also identified, such as α-Sn, the white matrix (P5 in Figure 3), and Sn–Zn solid solution, the grey structure (P6 in Figure 3b), according to the EDS results and the Zn–Sn phase diagram [46]. An island-like α-Zn solid solution phase (P7 in Figure 3d) was distributed at the FZ/Al interface. The strip-like black phase (P8 in Figure 3d) was identified as the α-Al solid solution. A thin banded layer adjacent to the Al base metal was inferred as the α-Al + η-Zn phase according to the chemical composition at position P9 in Figure 3d. 

From the direct irradiation region to the weld toe region at the interface, the thickness of the CuZn IMC layer gradually decreased from 5.45 μm to 3.45 μm due to the variation of the heat input, while the α-Zn solid solution content increased in the seam near the FZ/Cu interface. During the cooling and solidification of the seam, the flow and stirring of the molten pool were mainly concentrated in the weld toe zone. Hence, more α-Zn solid solutions were produced by more Zn atoms due to the flow of the molten pool in the weld toe zone. As the thickness of the tin foil increased from 0.3 mm to 0.5 mm, a similar microstructure was observed at the FZ/Cu interface (Figure 3e–h), and the thickness of the CuZn layer at the FZ/Cu interface decreased by approximately 0.3 μm. This decrease might have been caused by lower heat input and Zn atom content in the seam near the interface, which were both attributed to thicker tin foil as it hindered the flow of the molten Zn-based filler to the FZ/Cu interface. Consequently, the α-Zn solid solution content also decreased.

**Figure 4:**
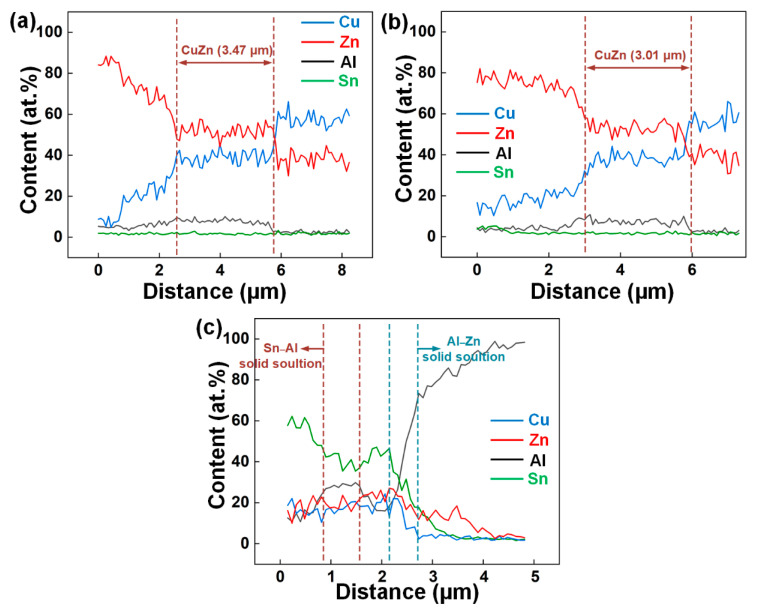
EDS line results in Figure 3: (**a**) at the FZ/Cu interface in Figure 3a; (**b**) at the FZ/Cu interface in Figure 3e; (**c**) at the FZ/Al interface in Figure 3h.

To further determine the element distribution, EDS line scanning analysis (Figure 4) was carried out in the weld toe zone at the FZ/Cu (Figure 3a,e) and FZ/Al interfaces (Figure 3h). The CuZn layer (thickness = 3.47 μm) is clearly observed in Figure 4a. Similar element distribution is observed in Figure 4b, but with lower thickness of the layer (3.01 μm). According to the elemental distribution shown in Figure 4c and the Al–Sn binary system [47], the white matrix phase in Figure 3h is possibly the Sn–Al solid solution. Compared with the Al/Cu lap joint with only the Zn–2Al weld filler [35,40], the interfacial microstructure changed because of the addition of the tin foil. The α-Sn and Sn–Zn solid solutions were produced near the FZ/Cu interface and the Sn–Al solid solution formed at the FZ/Al interface.

#### 3.2.2. Interfacial Microstructure of the Joint with Ni Coating

With the addition of the tin foil, a brittle CuZn IMC layer (thickness = 5.45 μm) was still generated at the FZ/Cu interface. Moreover, the addition of the tin foil led to the formation of α-Sn and Sn-Zn solid solutions near the FZ/Cu interface, which may negatively affect the bonding of the interface. Hence, Ni-coated copper sheets were used to limit the formation of Cu–Zn IMCs as the Ni coating on the Cu sheets could inhibit the diffusion of Cu. 

**Figure 5:**
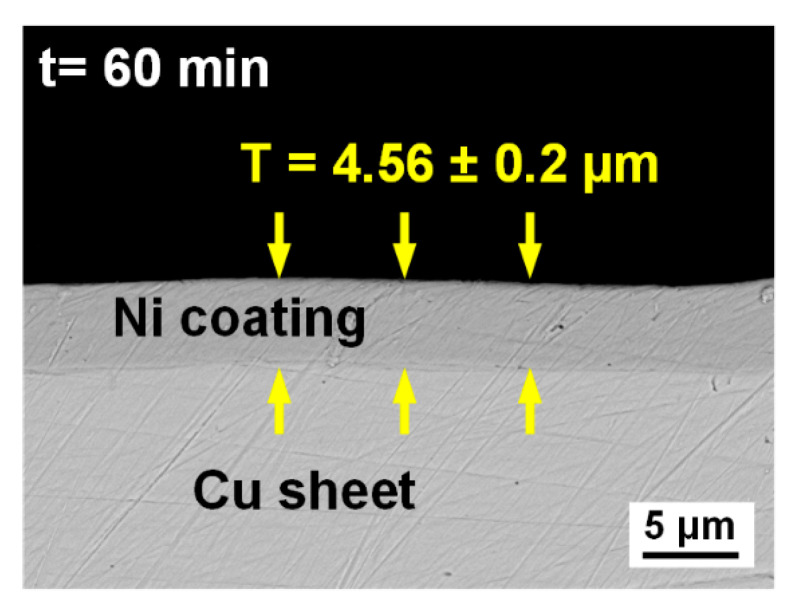
SEM images of the Ni coating at an electroplating time of 60 min.

Figure 5 shows the cross-sectional SEM image of the Ni-coated Cu sheet. Optimal thickness of the Ni coating, 4.56 μm, was obtained after an electroplating time of 60 min. The Ni coating was uniform and continuous, and the thickness error was controlled within 0.2 μm.

**Figure 6:**
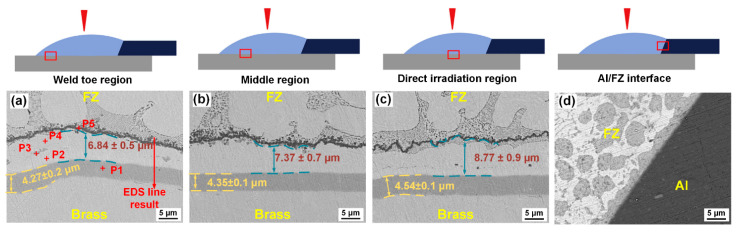
Microstructures of the FZ/Cu and FZ/Al interfaces: (**a**–**c**) FZ/Cu interface in the weld toe region, the middle region, and the direct irradiation region; (**d**) Al/FZ interface.

Figure 6 shows the micromorphology of four interfacial regions of the Al/Cu joint with a Ni-coated Cu sheet after the LWB process. The corresponding chemical compositions at different positions are listed in Table 5. A uniform and continuous layer (P1 in Figure 6a) is observed at the FZ/Cu interface. According to the EDS results at P1, this layer contained 0.4 at.% Al, 5.45 at.% Cu, 4.49 at.% Zn, and 91.55 at.% Ni. The melting point of Ni (1728 K) is higher than that of copper (1356 K). Hence, it can be inferred that the pre-plated Ni coating did not melt, and therefore, this layer (P1 in Figure 6a) is the Ni coating. The maximum Cu content was only 5.72 at.% at the FZ/Cu interface. Therefore, the low Cu content possibly inhibited the formation of CuZn IMCs. 

According to the EDS point results in Table 5, the inner layer (P2 in Figure 6a) adjacent to the Ni coating was identified as Ni_3_Zn_14_ IMCs based on the Ni–Zn phase diagram [48]. The outermost layer was a black zigzag phase (P5 in Figure 6a), and it contained 46.94 at.% Al, 29.89 at.% Ni, 20.92 at.% Zn, and 2.25 at.% Cu. Hence, in combination with the binary phase diagram, the phase was inferred as Al_3_Ni [49]. The Zn content of the layer (P4 in Figure 6a) sandwiched between Al_3_Ni and Ni_3_Zn_14_ was up to 88.74 at.%, indicating that this phase was the α-Zn solid solution. Some gray, small, block-like phase (P3 in Figure 6a) was sparsely distributed at the region between the Ni_3_Zn_14_ layer and the α-Zn solid solution. According to EDS results and the Al–Ni–Zn ternary phase diagram [50], the phase was identified as the τ_2_ Zn–Ni–Al ternary phase. 

**Figure 7:**
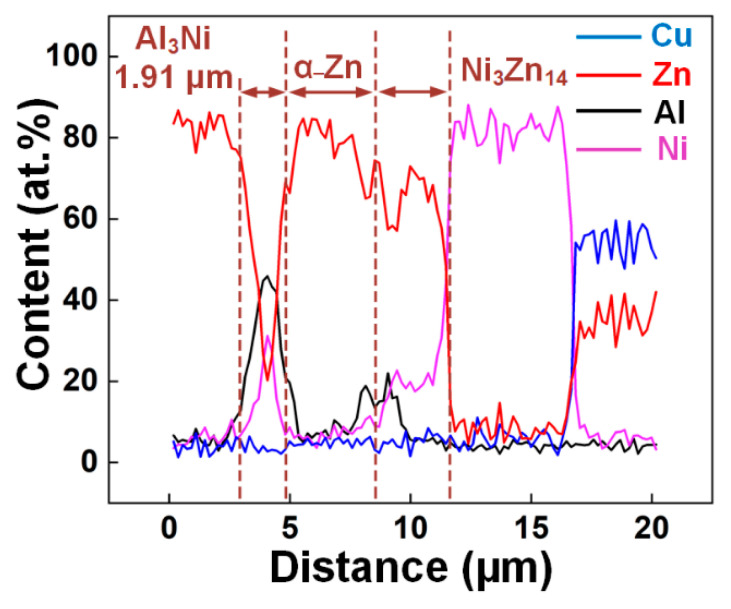
Line scanning result in Figure 6a.

As shown in Figure 6a, EDS line scanning analysis was carried out across the interface of the weld toe zone, and the corresponding EDS results are shown in Figure 7. The interfacial layers mentioned above were observed, of which the α-Zn solid solution layer was the thickest, and the thickness of Al_3_Ni was only 1.91 μm. In addition, the Al_3_Ni region had high Al content, and between the α-Zn solid solution layer and the Ni_3_Zn_14_ layer, Al was aggregated, which was attributed to the formation of the τ_2_ Zn–Ni–Al ternary phase. The Cu content in the seam was almost zero, and the diffusion of Cu was observed only in the Ni coating, demonstrating that the use of the Ni coating for hindering the diffusion of Cu was effective.

**Figure 8:**
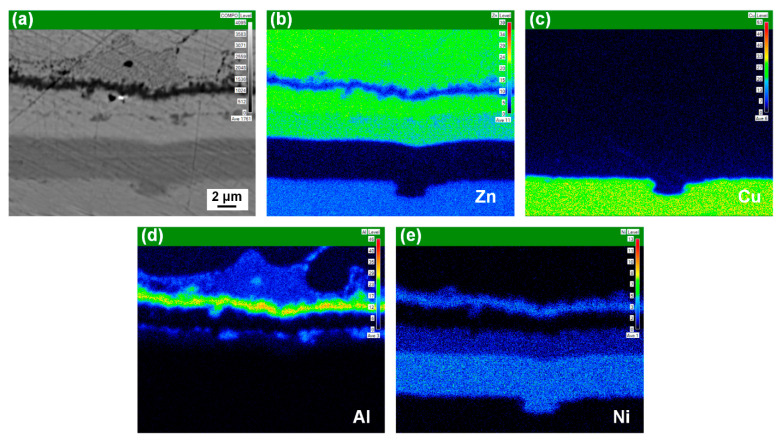
Map scanning results of the Al/Ni-coated Cu joint at the FZ/Cu interface: (**a**) initial microstructure of the FZ/Cu interface; (**b**–**e**) map scanning results of Zn, Cu, Al, and Ni.

The interfacial microstructure of the Al/Ni-coated Cu joint was complex. In addition to the three obvious layers, one additional layer also existed in the middle of the α-Zn solid solution layer and the Ni_3_Zn_14_ layer. To further determine the element distribution at the FZ/Cu interface, map scanning analysis was carried out across the interface. Figure 8 shows the elemental distribution of Zn, Cu, Al, and Ni in the weld toe region with the Ni coating (Figure 8a). Cu atoms in the copper substrate only slightly diffused to the seam due to the hindering effect of the Ni coating. As shown in Figure 8d, in addition to the Al_3_Ni IMCs layer, Al was also distributed in the network-like Al–Zn solid solution of the seam. In addition, Al was dispersed between the α-Zn solid solution layer and the Ni_3_Zn_14_ IMC layer, revealing the existence of the τ_2_ Zn–Ni–Al ternary phase layer, which was consistent with the results of micromorphology and line scanning at the interface. 

From the Cu base metal to the FZ, the phases produced were in the following order: Ni_3_Zn_14_, τ2 Zn–Ni–Al ternary phase, α-Zn solid solution, and Al_3_Ni.

### 3.3. Mechanical Properties

**Figure 9:**
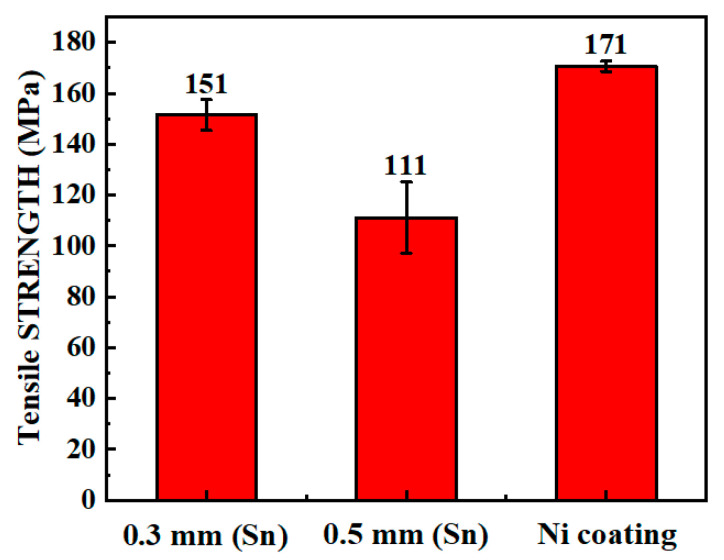
Tensile strengths of various joints.

Tensile strength tests were carried out on the samples using the Zn–2Al filler with the tin foil or the Ni coating. Figure 9 shows the tensile strengths of the various joints. In our previous work, the tensile strength of the joint with only a Zn–2Al filler was 148 MPa [40]. The average tensile strength of the joint with 0.3 mm tin foil was 151 MPa, which was not considerably higher than that without tin foil. A thicker tin foil caused a decrease in the strength, 111 MPa, which was lower than that of the joint without tin foil. As mentioned in the discussion on macroscopic morphology, the joint with a 0.5 mm thick tin foil had more defects such as incomplete fusion and depressions in the middle of the seam due to excessive penetration, which seriously affected the joint strength. The nonuniform morphology of the seam considerably affected the tensile strength, representing a larger error bar.

The joint with the Ni coating was significantly strengthened. The tensile strength increased to 171 MPa due to the enhanced interfacial bonding. Addition of the Ni coating was the most effective way to strengthen an Al/Cu joint with the Zn–2Al filler. 

**Figure 10:**
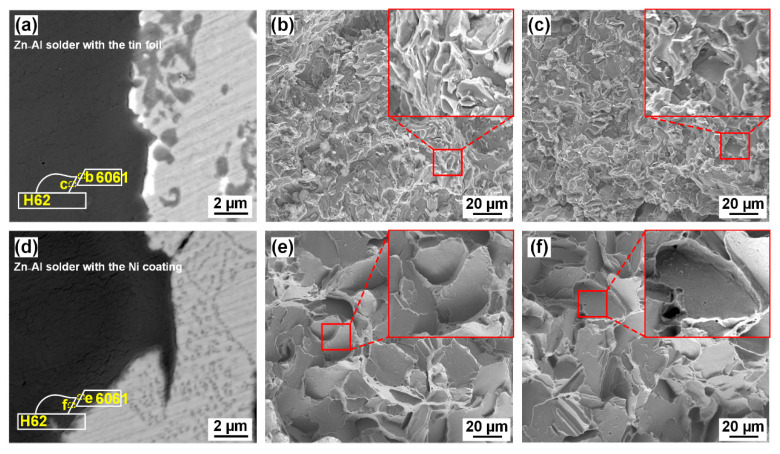
Fracture paths and fracture surfaces of the Al/Cu joints: (**a**) fracture path of the joint with the tin foil; (**b**) fracture surface of the joint with the tin foil at the Al sheet side; (**c**) fracture surface of the joint with the tin foil at the seam side; (**d**) fracture path of the joint with the Ni coating; (**e**) fracture surface of the joint with the Ni coating at the Al sheet side; (**f**) fracture surface of the joint with the Ni coating at the seam side.

Figure 10 shows the fracture paths and the micromorphology of the fracture surfaces, in which the specimens with various interlayers had the highest strength. Figure 10a,d shows the fracture paths of the joints with the tin foil and the Ni coating, respectively. Both joints fractured at the seam near the FZ/Al interface, indicating the enhancement of the strength at the FZ/Cu interface. Figure 10b shows that the fracture morphology of the joint with the tin foil was fine depressions. Studies have reported that the CuZn IMC layer at the FZ/Cu interface is often the weak area in Al/Cu joints [7,35,40]. The tin foil did not inhibit the formation of CuZn IMCs, but the spreading/wetting ability of the filler in the presence of the tin foil was considerably improved. Using a 0.3 mm tin foil, the weak area was transferred to the seam near the FZ/Al interface. According to the EDS line results in Figure 4c, the tin foil melted and flowed to the region close to the Al base metal, and the low strength of the Sn solid solution weakened the joint at the Al side. Hence, the strength increased slightly with the tin foil.

With the Ni coating, the fracture surfaces shown in Figure 10e,f exhibit shallow depressions and large smooth cracks, indicating more brittle characteristics.

Thus, addition of the tin foil and the Ni coating strengthened the joints, while the tensile strength was limited by the bonding near the Al sheet. 

### 3.4. Thermodynamic Analysis

As mentioned above, addition of the tin foil did not improve the interfacial bonding. Instead, the increased thickness of the CuZn IMC layer was detrimental to the interfacial bonding. The main reason for the increase in joint strength was that the tin foil improved the spreading/wetting ability of the molten solder. The interfacial microstructure of the joint with the Ni coating was complex, and it replaced the Cu–Zn IMCs. The results of the mechanical tests showed that using the Ni coating enhanced the FZ/Cu interfacial bonding. Hence, only the effects of the Ni coating were examined in this study. The interfacial reaction mechanism was further analyzed using thermodynamic calculations.

It is difficult to measure the temperature field of the FZ/Cu interface directly. Researchers often use numerical simulations to discern the thermal cycle at the interface. In our previous research [40], when a Zn–2Al weld filler was used with Al/Cu joints, the thermal cycle of three selected regions at the FZ/Cu interface was determined, and the average peak temperature of 838 °C was given as input to the Toop model to obtain the thermodynamic results. The Ni coating was very thin and the macromorphology of the joint was almost the same as that with only the Zn–2Al filler; therefore, its thermal effect was ignored in this study. Hence, the same interfacial peak temperature of 838 °C was used in the thermodynamic calculation even after the Ni coating was added.

Based on the Al–Zn–Cu ternary phase diagram [51], CuZn_5_ and CuZn could be produced at 598 °C and 468 °C, respectively. Ni_3_Zn_14_ and Al_3_Ni could be produced at 872–490 °C [52] and 850–643 °C, respectively [53]. The τ_2_ Zn–Ni–Al ternary phase could be formed at 850–340 °C [50], and the α-Zn and α-Al + η-Zn solid solutions could be formed at 419.6 °C and 381 °C [54]. All of the above phases can be produced at 838 °C.

In the Ni–Zn–Cu–Al quaternary system, to clearly describe the thermodynamic results, the data were divided into two stages. At the beginning of the welding process (stage I), the interface was assumed to be a 0.02Al–Zn–Ni–Cu quaternary system, with fixed Al content of 0.02 (same as the content of the Zn-based filler); this was used to analyze the diffusion tendency of Cu and Al atoms. Based on the distribution of Al and Cu, the subsequent reaction was analyzed, which was stage II. According to the EDS point results of the interfacial microstructure (Table 5), the Cu content in the seam was very small, with the maximum value of 0.057, suggesting that Cu did not participate in the interfacial reaction. Hence, the Cu content was fixed at 0.05 for the analysis of the 0.05Cu–Zn–Ni–Cu pseudoternary system. The molar fractions of Zn and Ni in the calculations were 0–0.95 each and those of Al were 0.05–0.997. 

**Figure 11:**
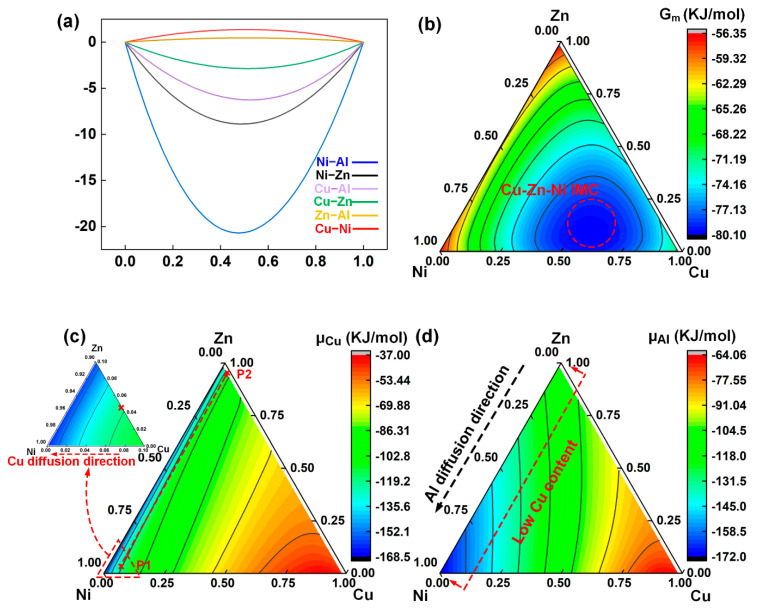
Thermodynamic calculation of the region at the interface of the Al/Ni-coated Cu joint in stage I: (**a**) formation enthalpy of binary system; (**b**) Gibbs free energy of the 0.02Al-(Ni-Zn-Cu) pseudo-ternary system; (**c**) and (**d**) chemical potential of Cu and Al in the 0.02Al-(Ni-Zn-Cu) pseudo-ternary system.

Figure 11 shows the thermodynamic calculation results of the 0.02Al–(Ni–Zn–Cu) pseudoternary system at 838 °C in stage I. Figure 11a shows the formation enthalpy in binary systems. Since the standard molar enthalpy is positive, Cu–Ni and Zn–Al systems cannot react with each other, but the metallurgical reaction can occur in the Ni–Al, Ni–Zn, Al–Cu, and Cu–Zn systems. The lowest formation enthalpy was observed for the Ni–Al system, implying that Ni–Al IMCs are easily produced. The Gibbs free energy of the 0.02Al–Cu–Zn–Ni system in stage I is shown in Figure 11b. Ni–Zn–Cu ternary IMCs with the lowest Gibbs free energy are easily produced in the system. However, the corresponding ternary IMCs were not observed at the FZ/Cu interface of the joints with the Ni coatings, which might be attributed to the changed Cu and Al content. Therefore, further analysis of the diffusion tendency of Cu was carried out, as shown in Figure 11c. According to the EDS results in Table 5, Cu content was very small in the seam near the Ni coating and 0.055 in the Ni coating, which might be attributed to the unmelted Ni coating. To clearly describe the diffusion tendency of Cu, the chemical potential gradient from P3 (Figure 11) to P4 (Figure 11) was calculated. Initial Cu content was set to 0.055 in the Ni coating (Table 5 P1), and the final Cu content was set to 0.036 in the α-Zn solid solution (Table 5 P4). The chemical potential gradient of Cu from the Ni coating to the α-Zn solid solution was −4.159 kJ/mol, as shown in Table 6, which suggested that the diffusion of Cu atoms was much more difficult when the Cu sheet was covered with the Ni coating. Figure 11d shows the chemical potential of Al atoms during stage I. With low Cu content, the Al atoms tended to diffuse from the molten filler with high Zn content to the Ni-enriched region. This led to the aggregation of Al atoms adjacent to the interface, which promoted the formation of Al–Ni IMCs. The final content of Al at the interface was much greater than 0.02, indicating that the previous assumption about the Al content (0.02) was incorrect. Hence, further analysis of stage II was carried out.

**Figure 12:**
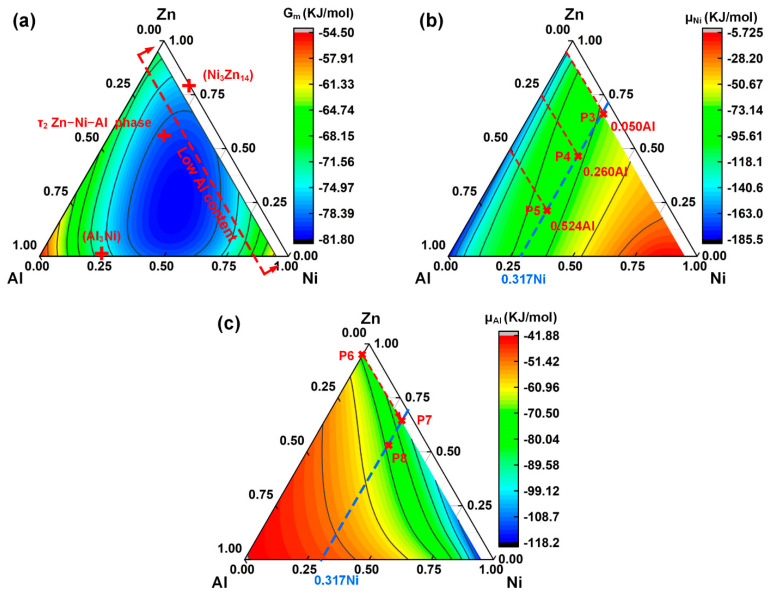
Thermodynamic calculations for various regions at the interface of the Al/Ni-coated Cu joint in stage II: (**a**) Gibbs free energy of the 0.05Cu-(Zn-Ni-Al) pseudo-ternary system formation enthalpy of binary system; (**b**) and (**c**) chemical potential of Ni and Al in the 0.05Cu-(Zn-Ni-Al) pseudo-ternary system.

Figure 12 shows the thermodynamic calculation results of stage II. To determine the preferentially generated phases at the interface, the Gibbs free energy of the 0.05Cu–Zn–Ni–Cu quaternary system was calculated, as shown in Figure 12a. The lowest standard Gibbs free energy of the system was observed in the blue area, implying that the Al–Ni–Zn ternary compounds might be the preferable phase produced. However, the actual phase generated at the FZ/Cu interface was not consistent with the inference, which resulted from the changing Al content. In the beginning, the Al content adjacent to the Ni-coated Cu sheet was only 2%. Then, Al atoms gradually gathered near the interface. Ni_3_Zn_14_ was produced preferentially when the Al content was low. According to the EDS results in Table 5, Al content in the Ni_3_Zn_14_ phase was almost zero. Hence, the preferable formation of Ni_3_Zn_14_ further resulted in the increase in the Al content at the interface. Thus, after the preferable formation of Ni_3_Zn_14_, the Al_3_Ni phase and the τ_2_ Zn–Al–Ni ternary phase containing 15.8 at.% Al were generated. 

Figure 12b shows the chemical potential of Ni in the 0.05Cu–Zn–Ni–Cu pseudoternary system. Ni tended to diffuse to the gathering area of Al and Zn, illustrating the mutual diffusion between Ni and Al. As mentioned above, the Al content increased gradually during the welding process. Hence, three chemical potential gradients of Ni were compared for the Al contents of 0.050, 0.260, and 0.524, and the Ni content was set to 0.317 based on the EDS point results in Table 5 (P5). The specific values of Ni chemical potential at P3, P4, and P5 (shown in Figure 12) were −70.14, −76.86, and −85.26 kJ/mol, as shown in Table 7. The order of chemical potentials at these three points was P3 > P4 > P5, indicating that Ni tended to diffuse to the region with a higher Al content. Thus, the unique diffusion phenomenon of Ni can be described. At the beginning of the welding, the content of Al was low, the Ni atoms gathered at the FZ/Cu interface, and the Ni_3_Zn_14_ phase was preferentially produced. Then, as the Al content increased, most of the Ni atoms diffused to the Al-aggregating region, and the Al–Ni IMCs formed.

The highest Al content near the interface was 0.469 (EDS results shown in Table 5), which was much higher than the Al content of the Zn–2Al filler. To determine the diffusion tendency of Al, the chemical potential of Al in the 0.05Cu–Ni–Zn–Al system was calculated, as shown in Figure 12c. Al contents at P6 and P7 were set to 0.051, and the Ni contents at P6 and P7 were set to 0.001 and 0.317, respectively. The chemical potential gradient of Al from P6 to P7 (−13.76 kJ/mol, as determined from Table 7) in Figure 12 indicates the diffusion tendency of Al from the molten filler to the FZ/Cu interface. Thus, Al diffused to the interfacial region with high Ni content. The chemical potential of P8 in Figure 12c was −72.47 kJ/mol, which was nearly the same as that of P6, suggesting that the Al content increased until the Al content reached 0.155 when the highest Ni content was 0.317. Hence, the Al content was limited, which inhibited the formation of the brittle Al_3_Ni phase. The final interfacial microstructure showed that the Al_3_Ni IMC content was low, and the thin zigzag Al_3_Ni IMC layer had little effect on the bonding of the interface.

### 3.5. Bonding Mechanism of the Al/Ni-Coated Cu Joint 

**Figure 13:**
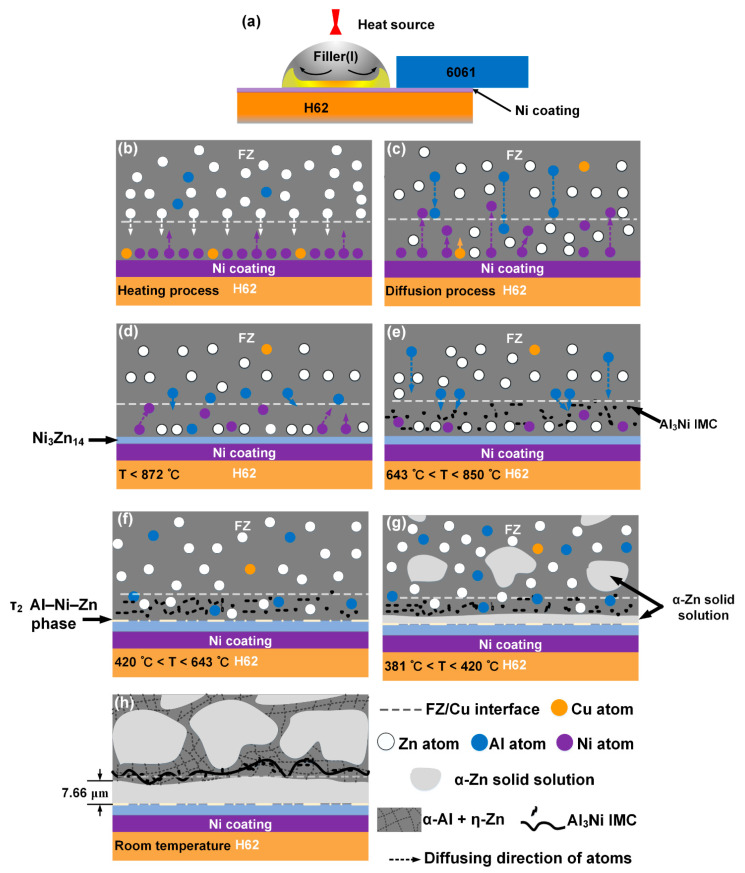
Schematic of the bonding mechanism at the interface of the Al/Ni-coated Cu joint: (**a**) schematic of the bonding; (**b**) heating process; (**c**) diffusion process; (**d**–**g**) cooling process with various temperature intervals; (**h**) final conditions of the Al/Ni-coated Cu joint at room temperature.

Based on the microstructure analyses and thermodynamic calculations of the Al/Ni-coated Cu joint, the formation of phases and the diffusion tendency of atoms at different temperature intervals are illustrated in Figure 13. Once the LWB process began, the Zn–2Al filler melted and rapidly spread on the surface of the Ni-coated Cu sheet, as shown in Figure 13a. Then, the interfacial temperature increased rapidly due to the spreading of the molten filler. In the heating process, as shown in Figure 13b, atoms from the Ni coating and the molten filler diffused toward each other due to the concentration gradient. As the temperature increased, the Ni atoms gathered at the region adjacent to the FZ/Cu interface, the Al atoms diffused from the molten filler to the Ni-gathering region, and few Cu atoms diffused to the seam, as shown in Figure 13c. In this diffusion process, the diffusion direction and the driving force depended on the chemical potential of the system. According to the aforementioned analysis, when the temperature decreased to 872 °C, the Ni_3_Zn_14_ layer was formed adjacent to the Ni coating due to the gathering of large quantities of Ni and Zn atoms, as shown in Figure 13d. Notably, the thickness of the Ni_3_Zn_14_ layer increased in a large temperature range of 872–490 °C. Due to the interdiffusion of Al and Ni atoms, the Al content gradually increased, and a gathering area of Ni and Al atoms formed close to the Ni_3_Zn_14_ layer, which resulted in the formation of black diamond-shaped Al_3_Ni particles in a temperature range of 850–643 °C, which dispersed in the seam near the Ni_3_Zn_14_ layer, as shown in Figure 13e. As the temperature decreased to 643–420 °C, a small amount of the τ_2_ Zn–Ni–Al ternary phase formed adjacent to the Ni_3_Zn_14_ layer, as shown in Figure 13f. Then, α-Zn solid solution formed when the temperature decreased to 420 °C, as shown in Figure 13g. Finally, the thickness of the α-Zn solid solution increased to an average of 7.66 μm. Due to the force of the solid front of the α-Zn solid solution layer around the Ni_3_Zn_14_ layer, the diamond-shaped Al_3_Ni phase aggregated, and finally, the zigzag Al_3_Ni layer generated around the α-Al + η-Zn solid solution adjacent to the α-Zn layer. As shown in Figure 13h, at room temperature, composite layers of the Ni_3_Zn_14_–τ2 Zn–Ni–Al ternary phase–α-Zn solid solution–Al_3_Ni were produced at the interface instead of the CuZn–CuZn_5_ layers without the Ni coating, which strengthened the interfacial bonding.

## 4. Conclusions

1. Due to addition of the tin foil, the spreading/wetting ability of the Zn–2Al filler on the Cu sheet considerably improved. With the 0.3 mm tin foil, the spreading width of the Zn–2Al filler increased to 8.54 mm and the contact angle was 46.74°, and the filler showed high wetting/spreading ability. While the tin foil weakened the bonding at the FZ/Al interface, it increased the strength of the joint slightly. In addition, large thickness of the tin foil caused excessive penetration, which negatively affected the tensile strength.
2. At the FZ/Cu interface of the Al/Cu joint with the tin foil, a brittle CuZn IMCs layer was observed adjacent to the Cu sheet, which was detrimental to the interfacial bonding. For the Al/Ni-coated Cu joint, composite layers of Ni_3_Zn_14_–τ2 Zn–Ni–Al ternary phase–α-Zn solid solution–Al_3_Ni formed at the interface adjacent to the Cu sheet.
3. Thermodynamic calculations revealed that the diffusion of Cu was greatly limited due to addition of the Ni coating. The reaction system could be regarded as a 0.05Cu–Zn–Ni–Al pseudoternary system due to the extremely low Cu content and the interdiffusion between Al and Ni atoms. The formation of phases was in the order Ni_3_Zn_14_ → Al_3_Ni → τ2 Zn–Ni–Al ternary phase → α-Zn solid solution → α-Al + η-Zn.
4. The Al/Cu joints with the tin foil and the Ni coating both failed at the seam near the FZ/Al interface. The tensile strength of the Al/Cu joint was 148 MPa without the tin foil, while the average strengths of the joints with the tin foil and the Ni coating were 151 MPa and 171 MPa, respectively. The tensile strength increased by up to 15.5% with the Ni coating due to the strengthening effect on the bonding at the FZ/Cu interface.

## Figures and Tables

**Table 1:** Chemical compositions of the base metals and the Zn-based filler (wt.%) [7,36].

	Cu	Al	Zn	Si	Cr	C	Mn	Fe	Mg	Ni
H62 Cu alloy	60.5–63.5	0.5–1.5	Bal.	<0.005	0.1	–	–	<0.15	–	–
6061 Al alloy	0.19	Bal.	0.04	0.71	0.08	1	0.15	0.35	–	–
Zn–2Al weld filler	0.25	1.85	Bal.	–	–	–	–	0.8	0.1	0.13

**Table 2:** Process parameters in this study.

Experimental Parameters	Value
Distance from the focus position to the Cu surface	+20 mm
Laser power	2400 W
Velocity of welding	0.36 m/min
Flow rate of the shielding gas, Ar	15 L/min
Velocity of wire feeding	4 m/min
Thickness of the tin foil	0.3/0.5 mm
Thickness of the Ni coating	4.56 μm

**Table 3:** Element parameters used in Miedema’s and Toop models [31,40,41,42].

Element	*T_m_*/K	*n_ws_*/d.u.	*φ*/V	*μ*	*V*/cm^3^
Cu	1356	3.18	4.55	0.07	7.72
Al	933	2.7	4.2	0.04	10
Zn	693	2.3	4.1	0.07	9.23
Ni	1726	5.36	5.26	0.04	6.6

**Table 4:** Compositions of different positions in Figure 3.

Position	Al	Cu	Zn	Sn	Possible Phases
1	10.74	41.87	46.77	0.62	CuZn
2	2.91	7.07	88.7	1.31	α-Zn solid solution
3	3.92	6.37	89.17	0.53	α-Zn solid solution
4	76.29	3.32	16.55	3.85	α-Al + η-Zn
5	2.46	5.11	9.35	83.08	α-Sn
6	1.48	10.24	33.32	54.96	Sn–Zn solid solution
7	2.96	3.91	91.68	1.45	α-Zn solid solution
8	91.68	3.91	6.14	0.42	α-Al solid solution
9	77.38	2.12	13.25	7.25	α-Al + η-Zn

**Table 5:** Compositions of different positions in Figure 6.

Position	Al	Cu	Zn	Ni	Possible Phases
1	0.4	5.45	4.49	91.55	Ni coating
2	0.44	3.14	59.07	37.34	Ni_3_Zn_14_
3	15.82	5.72	58.33	20.12	τ_2_ Zn–Ni–Al ternary phase
4	1.82	3.61	88.74	5.83	α-Zn solid solution
5	46.94	2.25	20.92	29.89	Al_3_Ni

**Table 6:** Chemical potential gradient at the indicated points in Figure 11 at 838 °C (molar fraction).

Point	Cu	Al	Zn	Ni	μ_Cu_ (kJ/mol)	△μ_Cu_ (kJ/mol)
P1	0.055	0.021	0.045	0.900	−121.144	−4.159
P2	0.035	0.021	0.910	0.055	−116.985	

**Table 7:** Chemical potential of Ni and Al at the indicated points in Figure 12 at 838 °C (molar fractions).

Point	Ni	Zn	Cu	Al	μ_Ni_ (kJ/mol)	μ_Al_ (kJ/mol)
P3	0.317	0.633	0.051	0.050	−68.394	–
P4	0.317	0.423	0.051	0.260	−74.055	–
P5	0.317	0.159	0.051	0.524	−81.132	–
P6	0.001	0.949	0.051	0.050	–	−72.769
P7	0.317	0.633	0.051	0.050	–	−86.533
P8	0.317	0.528	0.051	0.155	–	−72.466

## Data Availability

Data sharing is not applicable for this article.
